# Linking genome size variation to phenotypic selection on target traits

**DOI:** 10.1002/ecy.70442

**Published:** 2026-06-16

**Authors:** Lucrezia Laccetti, Emilio Petrone‐Mendoza, Donata Cafasso, Antonia Cristaudo, Fabio Pinheiro, Giovanni Scopece

**Affiliations:** ^1^ Department of Biology University of Naples Federico II Naples Italy; ^2^ Department of Biological, Geological and Environmental Sciences University of Catania Catania Italy; ^3^ Departamento de Biologia Vegetal Instituto de Biologia, Universidade Estadual de Campinas Campinas São Paulo Brazil

**Keywords:** abiotic and biotic selective pressures, *Dianthus*, floral traits, genome size, *Hadena* spp., leaf functional traits

## Abstract

Genome size (GS) is known to be highly variable among angiosperm species. However, this variation can also occur within species. Both interspecific and intraspecific variations in GS have been often found to be linked to phenotypic traits. Therefore, selective pressures acting on these target traits may indirectly shape GS evolution within and among species. However, the processes linking selective pressures to GS evolution are typically studied at broad phylogenetic scales, often overlooking how these processes operate at the microevolutionary level, where selection acts on standing variation within species. Recently diverging species or independently evolving lineages offer ideal settings to test whether selection shaping GS variation within lineages is reflected in patterns of GS variation among them, thereby linking microevolutionary and macroevolutionary processes. Here, we combined flow cytometric estimates of GS with measurements of leaf and floral traits, known to be targets of selective pressures, in both common garden and wild populations of two recently diverged *Dianthus rupicola* lineages. Then, we tested for an allometric relationship between GS and such phenotypic traits. Finally, we characterized the biotic and abiotic environment of wild populations and quantified plant reproductive success to identify selective pressures acting on traits showing an allometric relationship with GS. We found substantial GS variation, primarily driven by differences between the two lineages, but also occurring within lineages. GS showed a strong allometric relationship with two leaf traits, that is, stomata area and epidermal cell dimension, and one floral trait, that is, style length, in both lineages. Leaf traits reflected similar patterns of local adaptation to the edaphic environment in the two lineages, whereas divergent biotic pressures between lineages were associated with variation in style length. Our selection analysis revealed that style length was negatively associated with plant reproductive success in the lineage interacting with the pre‐dispersal seed predator *Hadena*, while the opposite trend was observed in the lineage where this interaction is absent. By demonstrating how ecological factors shape traits covarying with GS both within and between lineages, this study provides a valuable framework to bridge micro‐ and macroevolutionary processes in GS evolution.

## INTRODUCTION

Genome size (GS) is highly variable among extant angiosperms, ranging about 2400‐fold across species (Gregory et al., 2007; Hidalgo et al., 2017; Pellicer et al., 2018). This variation is not restricted to distant taxa, but can also occur among closely related and independently evolving lineages and even among populations of the same species (e.g., Biemont, 2008; Boutte et al., 2020; Pellicer et al., 2010; Šmarda et al., 2008; Terlević et al., 2022). GS diversity is largely attributed to differences in the accumulation and retention of noncoding repetitive sequences (Bennetzen & Wang, 2014; Wang et al., 2021) and whole‐genome duplication (WGD or polyploidy) events (Landis et al., 2018). Also, GS diversity may arise through errors in DNA repair and via dysploid chromosome number alteration during cell division (Schubert & Vu, 2016; Wang et al., 2021). Although these mechanistic processes underlying GS variation are relatively well characterized, less is known on the ecological and evolutionary processes working on this variation (Faizullah et al., 2021; Hessen et al., 2010). The majority of the literature has often emphasized genetic drift and neutral processes as primary drivers of GS evolution (Lynch & Conery, 2003; Whitney & Garland Jr., 2010). However, while drift often contributes to GS variation in angiosperms, it does not explain why GS distributions within large clades are heavily skewed and do not fit neutral expectations (Leitch & Bennett, 2004), or why different lineages frequently show GS reduction after polyploidy or TE bursts, implying an active removal or selection against excess DNA (Vu et al., 2015; Wendel et al., 2016). Also, a growing body of literature has shown that several phenotypic traits that are potential target of natural selection (e.g., growth rate, flowering time, flower size, leaf size) are associated with GS through an allometric relationship (Balao et al., 2011; Beaulieu et al., 2008; Meagher et al., 2005). Most of these relationships remain largely correlational, and the underlying causal mechanisms are still poorly resolved (e.g., Roddy et al., 2020). Some mechanistic links have been proposed, particularly through nucleotypic effects whereby GS can influence minimum cell size, cell packing density, and potentially developmental rates such as the time required to reach reproductive maturity (Balao et al., 2011; Kreiner et al., 2023; Roddy et al., 2020). Although the direction and strength of these relationships remain debated, such links have led to the hypothesis that variation in GS may contribute to phenotypic variation relevant for local adaptation (Kreiner et al., 2023; Stelzer et al., 2021). Together, these findings suggest that ecological selection in addition to neutral processes can play a key role in shaping GS evolution.

In line with this hypothesis, recent studies proposed that selection may act on plant functional traits (e.g., seed mass, stomatal dimension, xylem diameter) and floral traits (e.g., floral longevity, flower dimension) associated to GS, thus impacting its evolution (Beaulieu et al., 2008; Simonin & Roddy, 2018). For instance, selection favoring larger guard cells or increased stomatal size, traits linked to leaf gas exchange and water‐use efficiency, can indirectly drive increases in GS. Similarly, in a study conducted on *Silene latifolia*, flower size was found to be genetically correlated with GS and QTL mapping revealed overlapping regions influencing both flower dimension and GS (Meagher et al., 2005). These findings indicate that selection on flower size has the potential of indirectly affecting GS. Further, paleo‐physiological studies show coordinated shifts in GS with anatomical leaf traits (e.g., vein density, stomatal size), that align with changes in paleoclimate, implying that GS is associated with functional traits under different environmental conditions (Faizullah et al., 2021; Franks & Beerling, 2009; Simonin & Roddy, 2018).

Despite these advances, the above‐mentioned ecological drivers of GS variation have been mainly analyzed at broad phylogenetic levels (e.g., Beaulieu et al., 2008; Knight & Beaulieu, 2008), with few studies conducted at the microevolutionary scale (i.e., among populations of the same species; but see Dastpak et al., 2025; Roxo et al., 2023). This represents a critical gap, as microevolutionary studies are essential to understand how divergent environmental selection acting across populations may affect leaf and floral traits and indirectly GS evolution. This knowledge would help linking the well‐characterized macroevolutionary patterns to the underlying microevolutionary processes. In this context, recently diverged lineages provide an ideal framework, as they allow testing whether the mechanisms acting among populations within the same lineage also contribute to divergence between closely related taxa.

In this study, we focused on 11 populations of the *Dianthus rupicola* complex representing two independent lineages whose diversification started approximately 87 kya (Laccetti et al., 2026). Despite their high genetic similarity, these lineages are separated by strong reproductive barriers (Laccetti et al., 2026) and exhibit some phenotypic differences likely resulting from divergent adaptive trajectories.

The genus *Dianthus* is known for its extensive morphological diversity and large interspecific variation in GS (Balao et al., 2009; Franzoni et al., 2024; Terlević et al., 2022; Valente et al., 2010). Also, in this genus, GS variation has been shown to correlate with environmental gradients and both leaf and floral traits (Balao et al., 2011; Terlević et al., 2022). In particular, Balao et al. (2011) found, in the *D. broteri* complex, a correlation between GS and several leaf and floral traits predicted to be under abiotic and biotic selective pressures (e.g., stomatal size; calyx length). For instance, the length of the calyx or the corolla size is commonly found to be under positive selection in species pollinated by Lepidoptera (Alexandersson & Johnson, 2002; Soteras et al., 2020) and under negative selection in species attacked by antagonistic pre‐dispersal seed predators as the moths of the genus *Hadena* (Brothers & Atwell, 2014), two interactions typical of members of the genus *Dianthus* (Balao et al., 2024; Brothers & Atwell, 2014; Soteras et al., 2020). These findings raise the possibility that divergent selection on floral traits could drive GS differentiation, with biotic interactions playing a key, yet underexplored role.

We combined flow cytometric estimates of GS with measurements of leaf functional traits and reproductive floral traits in both common garden and wild populations. We further characterized the abiotic and biotic environments of natural populations and quantified plant reproductive success. This integrative approach allows us to test whether GS divergence among populations of the two lineages is consistent with selective pressures imposed by biotic and abiotic environmental conditions. Specifically, we asked the following questions: (1) What is the extent of variation in GS between and within the two closely related lineages? (2) Is there an allometric relationship between GS and phenotypic traits? (3) Are the traits showing an allometric relationship with GS targets of biotic and abiotic selective pressures? (4) Do these selective pressures explain GS divergence between and within lineages?

## METHODS

### Study species and study system

**FIGURE 1:**
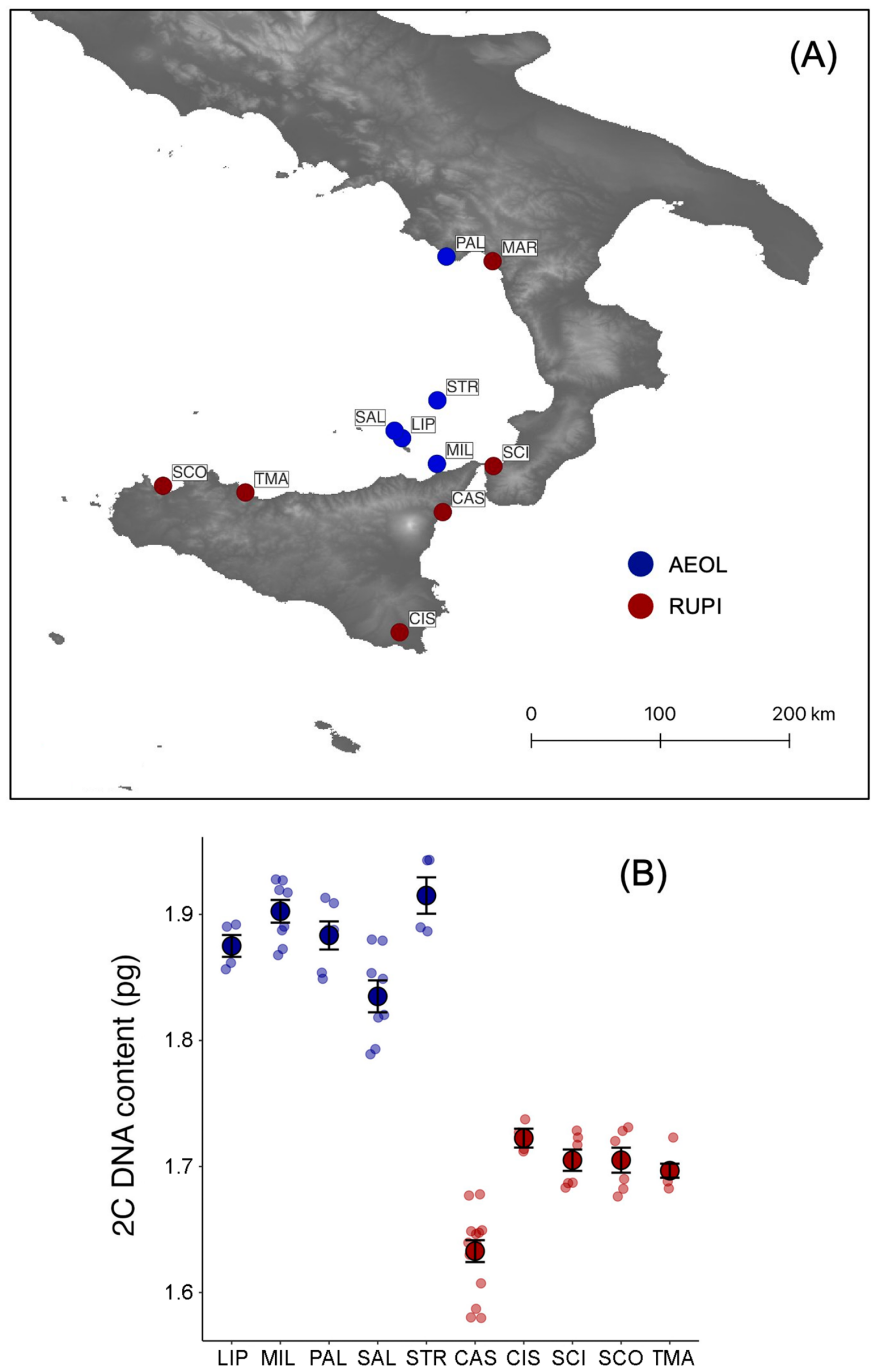
Geographic distribution (A) and genome size variation (B) of 11 *Dianthus rupicola* populations sampled in the study. Blue denotes the *aeolicus* lineage (AEOL) and red denotes the *rupicola* lineage (RUPI). Gray scale in the map denotes the elevation gradient in the study area with lighter colors indicating higher altitudes.

*D. rupicola* Biv. is a chasmophyte, strictly related to cliff habitats and endemic to the Central Mediterranean. The species flowers from early summer to early autumn and produces pink‐purplish flowers, aggregated in inflorescences. We investigated 11 populations located in peninsular southern Italy, Sicily and the Aeolian Islands, representing recently diverged lineages (Laccetti et al., 2026) with the same ploidy level (2*n* = 30; Peruzzi, 2003; Figure 1A; Appendix S1: Table S1). Experiments were conducted both in natural populations and on plants grown under common garden conditions. Seeds were collected from each natural population at the end of the 2020 flowering season and grown at the Department of Biology, University of Naples Federico II (Italy). Seeds from at least 10 maternal plants per population (interfamily distance ≥2 m) were collected within an area of approximately 250 m^2^ and germinated in greenhouses, producing 265 adult plants (*N*_min_ per population = 10, *N*_max_ = 20).

### GS estimation

We determined the absolute nuclear DNA content of 66 individuals from 10 *D. rupicola* populations (five populations from *rupicola* lineage [RUPI] and five populations from *aeolicus* lineage [AEOL]), by flow cytometry (FCM) with propidium iodide (PI) staining, using plants grown under common garden conditions. We choose this approach to overcome potential environmental biases that may create false variation among individuals (Biemont, 2008). To further minimize temporal and environmental effects on our GS estimates, individuals were randomly collected across a 3‐month period (September–December 2024). To test for measurement accuracy, on 30 individuals (15 for each lineage), we performed three replicates in different days. All the analyses were performed using fresh leaf material of approximately 0.5‐cm^2^ area. Samples were prepared following the instructions provided by the CyStain PI Absolute P kit. FCM measurements were taken using the CyFlow Ploidy Analyser (Sysmex Partec, Görlitz, Germany) equipped with a 532‐nm laser light source, at the Department of Biology of the University of Naples Federico II. The primary internal standard used to determine DNA amount was *Pisum sativum* “Ctirad,” a taxon with 9.09 pg of 2C nuclear DNA, as previously done in Balao et al. (2009). At least 3000 cells were analyzed in each measurement. Peak means and CVs were established through manual gating using FCS Express Version 7 De NOVO software (FCS Research, Los Angeles, CA, USA). We retained measurements with CV lower than 5%. The 2C values of the samples were calculated as the ratio between the 2C peak of the sample and the standard multiplied by the known GS of the standard. The range of GS variation within lineages was confirmed by performing simultaneous measurements of the most contrasting samples (e.g., Sliwinska et al., 2022; Šmarda et al., 2008; see Appendix S1: Figure S1). Lastly, we tested for the presence of endopolyploidy in leaf tissues, previously reported for other *Dianthus* species (e.g., Agulló‐Antón et al., 2013), on 10 plants (five per lineage), on three replicates per plant (Appendix S1: Figure S2). All the histograms provided were acquired using FlowJo v. 11.

### Floral trait measurements

To examine population divergence in floral traits, at the peak of the flowering period, we estimated, both in the natural populations and in the common garden, plant traits potentially attractive for pollinators and pre‐dispersal seed predators. For one population (SAL; Appendix S1: Table S1), we collected phenotypic traits only in the common garden. We recorded the number of flowers per inflorescence on 20 individuals and an average of 16 individuals in each natural population and each population grown in the common garden, respectively. Then, to account for variation within individuals, we collected six fully opened flowers (three flowers in the male phase and three flowers in the female phase) per individual in natural populations (*N* = 1080 flowers) and in the common garden (*N* = 1044 flowers). Each flower was photographed using a Sony alpha 65 digital camera and a millimeter scale as a reference. The software package Image J (https://imagej.nih.gov/ij/) was thus used to measure the following parameters: (1) style length, (2) calyx length, (3) petal length, (4) petal width, and (5) corolla diameter. Style length was determined as the distance from the nectary to the tip of the stigma.

### Leaf functional trait measurements

To investigate population divergence in leaf functional traits, we measured leaf area, leaf mass area (LMA), stomatal area, and epidermal cells area in plants from the common garden. We measured leaf area and LMA for 37 individuals (20 from RUPI and 17 from AEOL) from eight *D. rupicola* populations (four populations per lineage). For each individual, we collected 10 fully expanded leaves, except for three individuals where only five leaves were collected. To minimize shrinkage from rehydration, leaf area was measured within 24 h of collection using a flatbed scanner and ImageJ (https://imagej.nih.gov/ij/). Leaves were dried at 70°C for at least 48 h, and dry mass was determined with a gravimetric balance. LMA was calculated as dry mass divided by leaf area (in grams per square meter). For stomatal and epidermal measurements, one additional fully expanded leaf per individual was preserved in 70% ethanol until analysis. Epidermal peels were prepared from the abaxial surface and mounted in water for observation under a light microscope at 40× and 100× magnification (Motic, Hong Kong, China). Digital images were captured with a Motic camera attached to the same microscope using Motic software. Stomatal and epidermal cell sizes were measured with the Artificial Intelligence‐based software StomataPy (https://github.com/Alias-z/StomataPy), and erroneously identified cells were manually corrected using ISAT‐SAM software (https://github.com/yatengLG/ISAT_with_segment_anything).

### Characterization of the biotic environment

To characterize mutualistic interactions between plants and pollinators, in nine natural populations of *D. rupicola*, we characterized the pollinator community by recording pollinators' visits at the peak (i.e., late July) and at the end of the flowering season (i.e., late September). Observations were conducted during the potential peak of activity of different pollinator classes, that is, in the late morning (10:00–1:00 p.m.), in the early afternoon (4:00 p.m.–6:00 p.m.) and in the late afternoon (6:00 p.m.–9.00 p.m.). For each visit, we recorded the insect taxon and behavior. Pollinators were considered as all those insects landing on the flower and touching its reproductive structures. Pollinator data were recorded in 145 h of sampling. For all the populations, we estimated the relative contribution of each pollinator species as the ratio between number of visited flowers by a given species and total number of visited flowers.

To quantify the presence of pre‐dispersal seed predators of the genus *Hadena*, at the end of the flowering season, we recorded in nine populations, on 20 plants per population, the total number of fruits and the number of fruits showing the presence of *Hadena* larvae or of holes with a diameter higher than 1 mm on the side of the fruit. We used this threshold to distinguish between holes produced by larvae of *Hadena* and smaller holes produced by other sporadic pre‐dispersal seed predators. For taxonomic identification, larvae were fed on unripe seeds of *D. rupicola* until they pupated; the emerged moths were then used to identify the species. To verify if antagonistic selective pressures varied across flowering seasons, the same procedure was performed in three consecutive flowering seasons.

### Characterization of the abiotic environment

To characterize the abiotic environment of each population, we selected climatic and edaphic variables known to be ecologically relevant to *D. rupicola* distribution and linked to leaf functional traits. Climatic data at 30 arc‐sec (approximately 1 km) resolution were obtained from WorldClim v. 2.1 (http://worldclim.org/bioclim) and edaphic data at a resolution of 250 m were obtained from Soilgrid (www.SoilGrids.org). From the WorldClim database, we used five bioclimatic variables (see Appendix S1: Table S2) representing summaries of means and variations in temperature and precipitation and the variable elevation. Edaphic data included soil organic carbon (SOC), bulk density, cation exchange capacity (CEC), pH, coarse fragments, and N content. Climatic and edaphic data were extracted using the point sampling tool plugin in QGIS v. 3.16.15 (QGIS Development Team, 2021).

### Estimation of plant reproductive success

To estimate plant reproductive success, at the end of the flowering season, we counted the number of fruits from each of the 20 labeled plants in each natural population and collected three inflorescences from each plant, which led to a total number of 3886 fruits. We selected capsules ripe but not already open and recorded the seed number within each capsule. Then, we calculated the mean number of seeds per capsule and the total number of seeds per plant by multiplying the mean number of seeds per capsule by the total number of fruits produced by the plant.

### Statistics

All statistical analyses were performed in R 4.3.3 (R Core Team, 2024). Prior to analyses, all the traits were averaged per plant.

#### Quantifying genetically based divergence

To test for genetically based divergence in GS, floral traits and leaf functional traits, we used data from plants grown in the common garden. The divergence was tested by building generalized linear mixed models (GLMMs) with a Gamma distribution and log link function, as implemented in the R package *glmmTMB* (Brooks et al., 2017). All the models were built including lineage as a fixed effect and population as a random factor. Significance of the fixed effects was tested through the Type‐III Wald χ^2^ tests in the R package *car* (Fox & Weisberg, 2019). The distribution of GS variance as a result of differences between lineages, among populations and among plants within populations was determined using the R package *insight* (Lüdecke et al., 2019).

#### Testing the relationship between GS and phenotypic traits

To investigate the allometric relationship between GS and phenotypic traits in the two lineages, we used phenotypic data collected in the common garden. Specifically, we first applied Spearman Rank correlation tests between GS and phenotypic traits within each lineage. Then, following Jiang et al. (2026), scaling relationships between GS and significantly correlated phenotypic traits were tested using the standard major axis regression, as implemented in the R package *smatr* (Warton et al., 2012). Slope tests and elevation tests were conducted using the same R package. Lastly, to identify which trait carries the strongest and most consistent association with GS, we computed partial Spearman correlations between GS and each trait, controlling for the other two separately for each lineage.

#### Testing the relationship between GS and environmental factors

To test the relationship between GS and biotic and abiotic factors, we used correlation analyses as described for phenotypic traits. Before performing the analyses, abiotic factors (climatic and edaphic variables) were reduced into a set of orthogonal axes using a principal components analysis (PCA) in the *factoMineR* R package (Husson et al., 2016). We then used the scores of the first three principal components (PCs) to perform subsequent analyses. The correlation between GS and biotic factors was tested using the relative contribution of the dominant pollinator and the pre‐dispersal seed predator *Hadena*. As *Hadena* was only observed in the RUPI lineage, its effect on GS was analyzed separately. Putative correlations between abiotic and biotic factors were tested using Kendall rank correlation in the *PerformanceAnalytics* (Peterson et al., 2018).

#### Investigating signals of local adaptation in wild populations

To test for signals of adaptation to the biotic and abiotic environment, we used biotic and abiotic data obtained as described above. First, generalized linear models (GLMs) were used to test the association between abiotic factors and leaf functional traits showing an allometric relationship with GS. These models were built including leaf traits as response variables and abiotic factors as fixed effects. Similarly, GLMs were used to test the association between biotic factors and floral traits. As the presence of the nursery pollinator *Hadena* was observed only in one lineage (RUPI), a separate model testing the effect of *Hadena* on floral traits was used for RUPI.

#### Assessing the action of selection in wild populations

To specifically test the hypothesis that antagonists exert selection on floral traits, we estimated selection gradients in each wild population by using Path Analysis (PA). We could not include GS in these analyses because data were only collected on plants grown in the common garden. Selection can be either the result of a direct effect of a plant trait on reproductive success or an indirect effect mediated by biotic interactions (Scheiner et al., 2000). Total direct selection is the sum of the path coefficients of direct and indirect effects. As described above, we built separate models for the two lineages. In both models, we included floral traits that were found to be associated with GS and plant reproductive success, estimated as the total number of seeds per individual. We hypothesized that flower dimension was positively associated with the percentage of predation by *Hadena* due to a higher plant attractiveness (Brothers & Atwell, 2014). Also, due to the hypothesis that the interaction with *Hadena* is mainly antagonistic, we predicted that floral dimension was negatively associated with plant reproductive success, both directly and indirectly through the action of *Hadena*. Differently, in the lineage where *Hadena* is absent we hypothesized that flower dimension was under positive selection and thus that it was positively associated with reproductive success. This is based on previous studies reporting that Lepidoptera usually selects for larger floral displays (e.g., Kelber, 1997). These traits enhancing pollinator attractiveness are likely to be under positive selection in absence of *Hadena*. The analyses were performed using the *lavaan* R package (Rosseel, 2012).

## RESULTS

### GS estimation

According to the FCM measurements, the amount of 2C DNA ranged between 1.58 and 1.94 pg. The coefficient of variations for the fluorescence peaks ranged between 1.3% and 4.8% (4.01% ± 1.89 SD), and thus, all the measurements were retained. Measurement error, calculated as the SD of the three replicates per individual, was lower than 0.01 in all the cases. The amount of 2C DNA varied on average 1.05‐fold within populations, 1.10‐fold among populations within the RUPI lineage, 1.08‐fold among populations within the AEOL lineage, and 1.22‐fold between lineages. The variation in GS within lineages was further confirmed by concurrent FCM runs which resulted in distinct fluorescence peaks or bimodal distributions (Appendix S1: Figure S1). All the populations of AEOL showed a significant increase in GS compared to RUPI populations (χ^2^ = 95.484; df = 1; *p* < 0.001; Figure 1B). Variance partitioning analysis showed that the difference between lineages explained 85.30% of the total variance, while among‐ and within‐population differences explained 7.7% and 7%, respectively. We did not detect evidence of endopolyploidy in leaf tissues of the two lineages, which only contained 2C and a small amount of 4C nuclei, thus indicating that these tissues maintain the diploid level (Appendix S1: Figure S2).

### Phenotypic traits and covariation with GS

**FIGURE 2:**
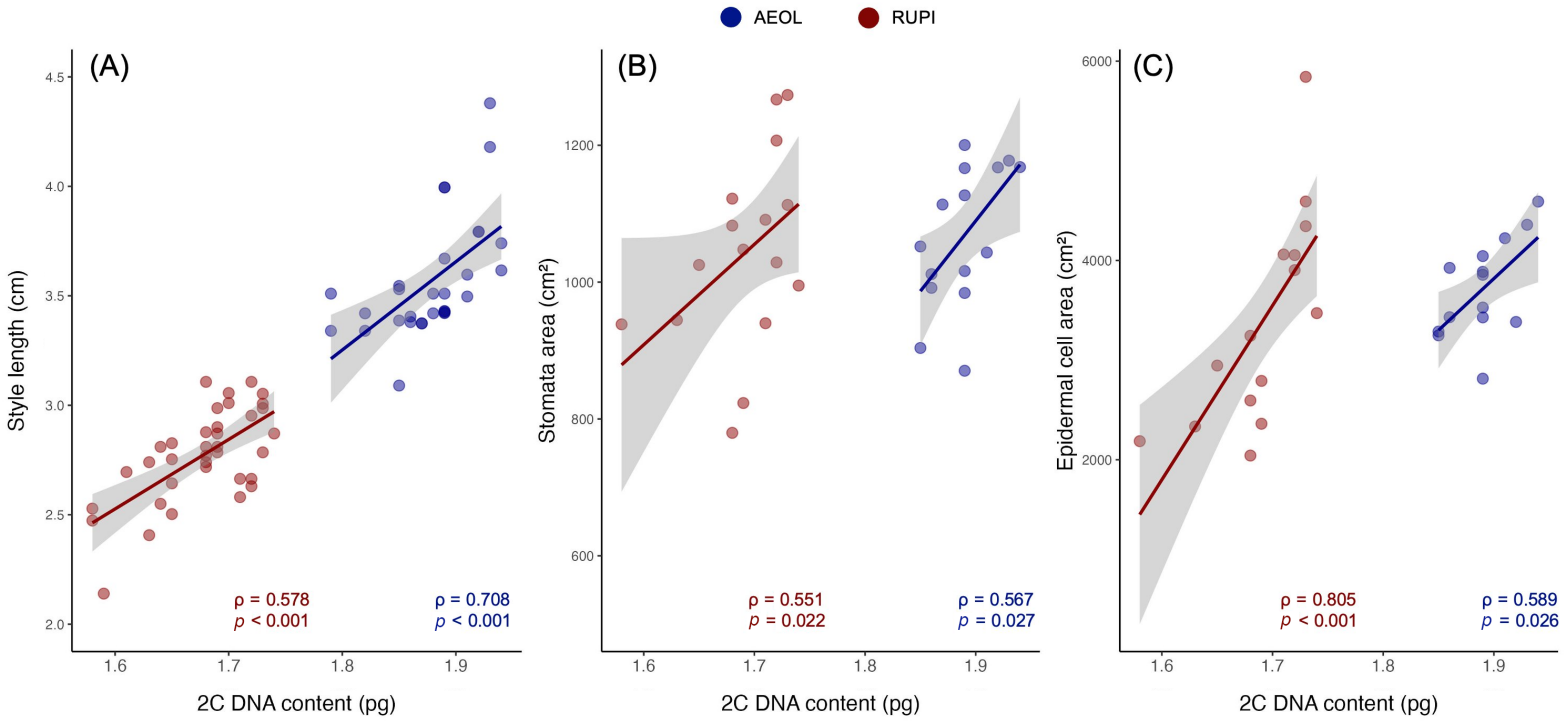
Covariation between genome size, floral traits (A) and leaf functional traits (B, C) in *Dianthus rupicola*. Blue denotes the *aeolicus* lineage (AEOL) and red denotes the *rupicola* lineage (RUPI).

All the investigated floral traits showed a significant difference between the lineages and a low among‐population variability (Appendix S1: Table S3, Figure S3). By testing the covariation between floral traits and GS, we observed a significant correlation with style length, while all the other traits did not vary with GS (Figure 2; Appendix S1: Table S4). Among leaf functional traits, a significant difference between the lineages was observed in leaf area (Appendix S1: Table S3, Figure S4), while no significant differences were observed in epidermal cell area, stomata area, and LMA (Appendix S1: Table S3, Figure S4). Among these traits, epidermal cell area and stomata area were significantly correlated with GS, while no significant correlation was observed between leaf area and LMA and GS (Figure 2; Appendix S1: Table S4). Slopes did not differ significantly between lineages for the two leaf traits (epidermal cell area: *r* = 1.176, df = 1, *p* = 0.278; stomatal complex area: *r* = 0.004, df = 1, *p* = 0.952), while we observed a marginal tendency towards divergence in the relationship between GS and style length (*r* = 3.007, df = 1, *p* = 0.083). In contrast, elevations differed significantly for all traits (epidermal cell area: *t* = 32.66, *p* < 0.001; stomatal complex area: *t* = 32.26, *p* < 0.001; style length: *t* = 7.03, *p* = 0.008), revealing that, at any given GS value, trait values are systematically shifted between the two lineages. Also, style length showed a significant partial correlation with GS in both lineages (AEOL: ρ = 0.709, *p* = 0.010; RUPI: ρ = 0.639, *p* = 0.019).

### Characterization of the biotic and abiotic environment

We recorded a total of 2750 pollinators' visits that revealed a different pollinator assemblage among populations of the two lineages (Appendix S1: Figure S5). These differences were consistent across the flowering season with no variation in the pollinator assemblage in none of the populations. Specifically, the hummingbird hawk–moth *Macroglossum stellatarum* was the main pollinator in all the populations. *Gonepteryx cleopatra* was observed in one population of RUPI and one population of AEOL. *Thymelicus lineola* and *Amegilla quadrifasciata* were, instead, observed only in RUPI, while *Gegenes pumilio* was observed only in AEOL. By analyzing antagonistic interactions, we detected the presence of two moths of the genus *Hadena*, that is, *Hadena bicruris* and *Hadena compta*, that act as nursery pollinators and pre‐dispersal seed predators, only in populations of RUPI. The frequency of this interaction was however variable among populations. In particular, the two populations where both *Hadena* species were recorded, that is, CMO and SCI, showed a percentage of seed predation of 28.21% and 20.24%, respectively, while SCO and TMA, where only *H. compta* was found, showed a percentage of seed predation of 14.04% and 23.53%. Comparable results were observed across the three investigated flowering seasons (Appendix S1: Table S6). The first three PCs explained 83.96% (PC1 = 29.68%, PC2 = 28.06%, PC = 26.22%) of variance in the abiotic environment (Appendix S1: Figure S6). The variables with the highest contribution along PC1 were MAT, MTWQ, and ELEV. Along PC2, PDQ, AP, and PWQ showed the highest contribution. SOC, bulk density, and N content were the variables most related to PC3.

### Correlation between GS and the biotic and abiotic environment

We observed no significant correlation between GS and abiotic factors (PC1, PC2, and PC3). The relative contribution of *M. stellatarum* was positively correlated with GS (ρ = 0.949, *p* = 0.051; ρ = 0.975, *p* = 0.005, in AEOL and RUPI, respectively). Within the RUPI lineage, the percentage of predation by the pre‐dispersal seed predator *Hadena* was negatively correlated with GS (ρ = −0.975, *p* = 0.005). We observed no significant correlation between abiotic factors and the relative contribution of *M. stellatarum* and *Hadena* spp. (Appendix S1: Figure S7).

### Signals of local adaptation and action of selection

**FIGURE 3:**
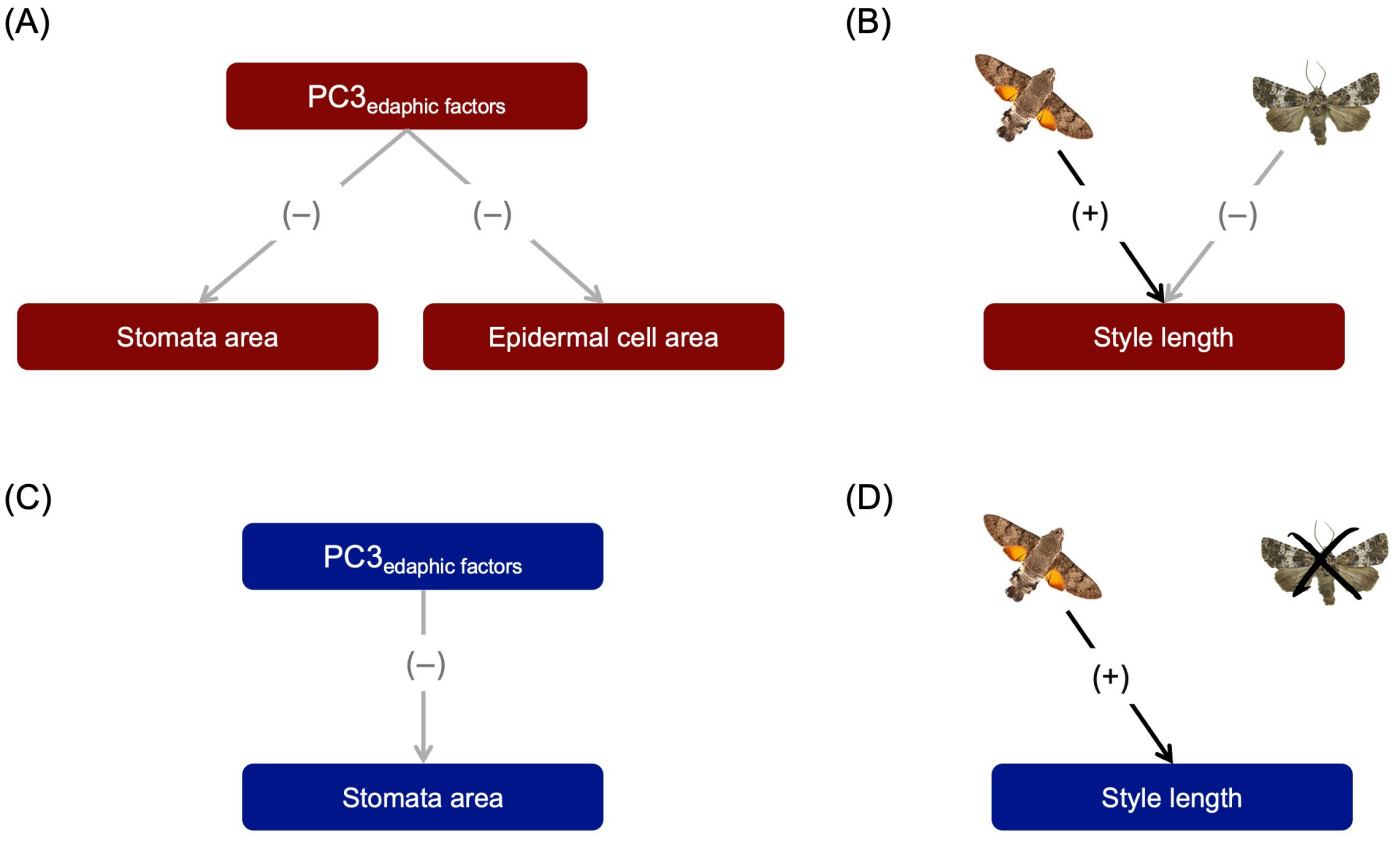
Signals of local adaptation to the abiotic and biotic environment in leaf functional traits and floral traits showing an allometric relationship with genome size. In red, the models performed within the *rupicola* lineage (A, B), in blue, the models performed within the *aeolicus* lineage (C, D). Black and gray arrows denote a positive and negative significant association (*p* < 0.05), respectively. Illustrations by Lucrezia Laccetti.

**FIGURE 4:**
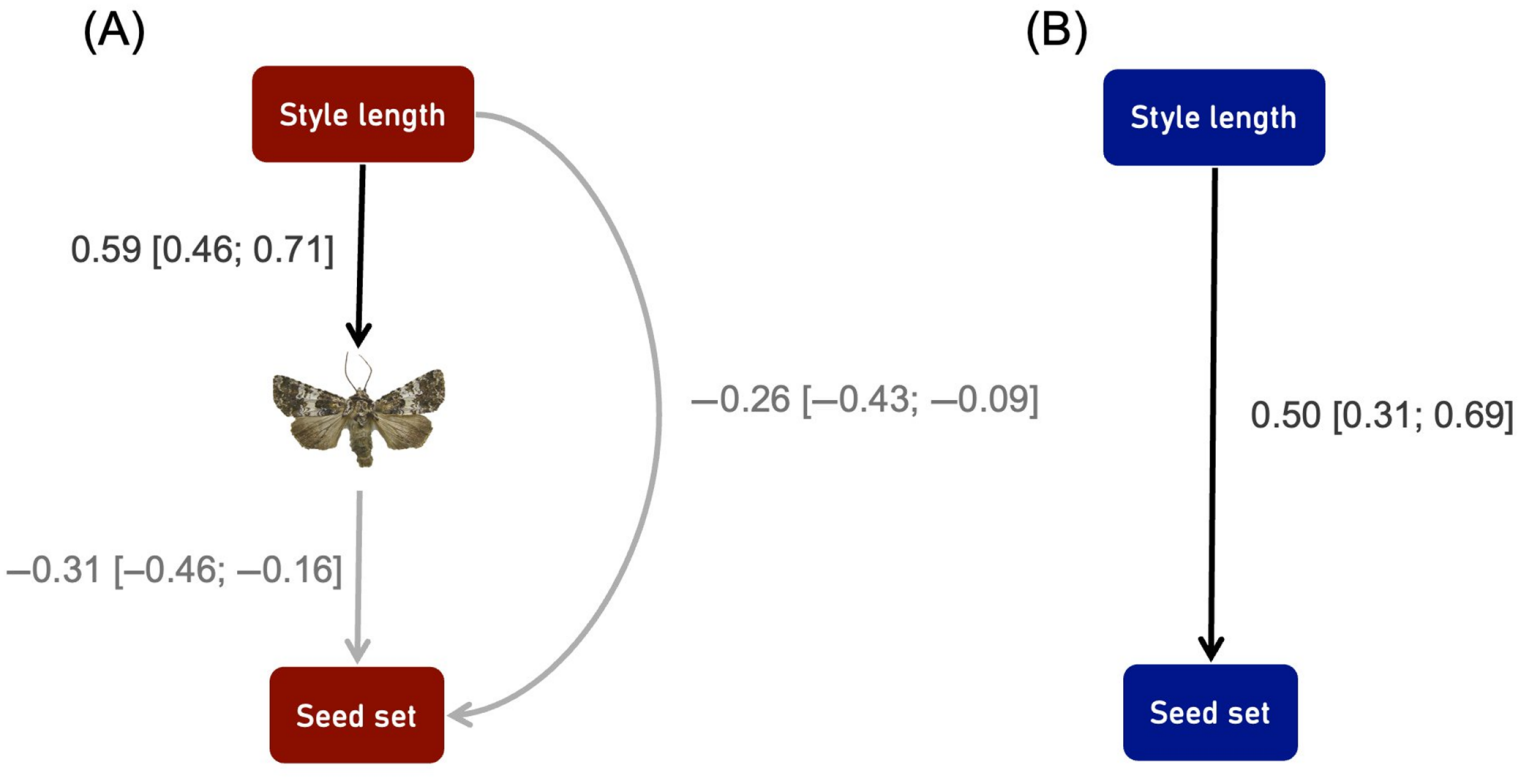
Diverging selective pressures acting within *Dianthus rupicola* lineages. Estimation of selection within the lineages obtained through structural equation models. In red, the model performed within the *rupicola* lineage including the nursery pollinator *Hadena* (A), and in blue, the model performed within the *aeolicus* lineage where *Hadena* is absent (B). Black and gray arrows denote a positive and negative effect (*p* ≤ 0.001), respectively, using bootstrap procedure with 1000 iterations. The 95th percentile bootstrap quantiles are reported in brackets. Illustrations by Lucrezia Laccetti.

We found a significant effect of PC3 (edaphic factors) on stomata area and epidermal cell area, while no significant effect was observed for PC1 (temperature) and PC2 (precipitation) (Figure 3; Appendix S1: Table S7). *M. stellatarum* had a significant positive effect on style length in both lineages and *Hadena* had a significant negative effect on style length in RUPI (Figure 3; Appendix S1: Table S7). We also observed a divergent selection acting on style length in the two lineages (Figure 4; Appendix S1: Table S8). The path analysis performed for the RUPI lineage yielded a final model where style length had a strong positive direct effect on the percentage of predation by *Hadena*, and a negative direct and indirect effect, mediated by *Hadena*, on plant reproductive success (Figure 4; Appendix S1: Table S8). Within AEOL, instead, we found a strong direct positive effect of style length on reproductive success (Figure 4; Appendix S1: Table S8).

## DISCUSSION

The processes linking selective pressures to GS evolution are typically studied at broad phylogenetic scales, often emphasizing abiotic drivers such as temperature, water availability, and altitude (Beaulieu et al., 2008; Díez et al., 2013; Knight et al., 2005). How these processes operate at the microevolutionary level, where selection acts on standing variation within species, is far less understood. In our study, we focused on the *D. rupicola* complex, which belongs to a genus known to harbor high GS variation (e.g., Balao et al., 2009; Franzoni et al., 2024; Terlević et al., 2022). By combining field and common garden data, we demonstrate that in two closely related *Dianthus* lineages, GS has an allometric relationship with leaf and floral traits that are targets of biotic and abiotic selective pressures, and that these relationships are reflected in trait differences between the lineages. Together, these results provide compelling evidence that microevolutionary processes driven by biotic and abiotic selective pressures can alter the distribution of GS between recently diverged lineages, linking microevolutionary and macroevolutionary dynamics.

Our survey of GS in 10 *D. rupicola* populations showed a substantial variation, mainly occurring between lineages (1.22‐fold; Figure 1). Interestingly, within each lineage we also found considerable variation among populations (1.10‐fold for RUPI, 1.08‐fold for AEOL) and, to a smaller extent, even within populations (an average of 1.05‐ and 1.03‐fold for RUPI and AEOL, respectively). This allowed us to test how different environmental conditions may act as selective agents on target phenotypic traits and indirectly on GS.

In both lineages, we observed that GS was positively associated with epidermal cell and stomatal area (Figure 2), consistent with the well‐established relationships between GS and such leaf functional traits (e.g., Beaulieu et al., 2008; Hodgson et al., 2010; Jiang et al., 2026; Simonin & Roddy, 2018). Similarly, GS showed a positive association with the floral trait style length (Figure 2), as previously reported in other plant species, including other species of the genus *Dianthus*, where floral dimensions correlate with nuclear DNA content (e.g., Balao et al., 2011; Cacho et al., 2021). One possible explanation for the observed relationships is the nucleotypic effect, whereby variation in GS may influence minimum cell size (Balao et al., 2011). Changes at the cellular level could in turn affect epidermal cell and stomatal dimensions and potentially scale up to organ‐level traits such as floral structures, although these links remain largely hypothetical and require further mechanistic investigation.

Interestingly, we observed that style length showed the most robust and consistent association with GS in the two lineages, suggesting that floral traits may represent a key ecological factor underlying GS evolution in our study system. Notably, our SMA analyses revealed significant differences in elevations (intercepts) between lineages for all traits, and a marginally significant difference in slope for style length, suggesting that the scaling relationships between GS and these traits differ between lineages, as reported in recent studies (e.g., Jiang et al., 2026). This pattern is consistent with the possibility that natural selection acts directly on these traits, potentially decoupling their evolution from GS and shaping among‐population variation in GS in *D. rupicola*.

Our findings revealed strong signals of local adaptation driven by abiotic and biotic selective pressures in phenotypic traits associated with GS (Figure 3). Specifically, we observed that edaphic conditions experienced by wild populations, rather than broader climatic gradients, had a significant effect on the mean population dimension of leaf functional traits and that this was consistent in the two lineages (Figure 3A,C). In particular, we observed that epidermal cell area and stomata area decreased their size with higher SOC and N content, and a lower bulk density. This finding suggests that, as previously reported, under high‐N conditions, plants may have prioritized rapid cell division over elongation, resulting in smaller, more responsive stomata (e.g., Drake et al., 2013; Oner, 2024).

This finding emphasizes the importance of fine‐scale soil heterogeneity in driving local adaptation, as demonstrated in the same study system (Laccetti et al., 2025) and in other Mediterranean plants (Veselý et al., 2020). Therefore, edaphic selection may represent a key abiotic mechanism maintaining GS diversity at microgeographic scales. Our findings show that within each lineage, soil heterogeneity selects for contrasting physiological and anatomical strategies that are linked to nuclear DNA content, thus suggesting that such heterogeneity likely explains the fine‐scale GS variation observed among populations of the same lineage.

Abiotic factors can also shape the biotic environment experienced by populations by affecting the abundance and composition of mutualists and antagonists (e.g., Burkle & Runyon, 2016; Soper Gorden & Adler, 2013). To test this, we examined whether climatic and edaphic gradients were associated with variation in the contribution of the dominant pollinator, the hummingbird hawk–moth *M. stellatarum*, and the pre‐dispersal seed predators of the genus *Hadena*. Contrary to expectations, we found no evidence of a direct link between abiotic and biotic factors, suggesting that pollinators and antagonists' contributions are shaped by other ecological constraints (Appendix S1: Figure S7). Nonetheless, biotic interactions had pronounced effects on a floral trait, style length, strongly correlated with GS (Figure 3B,D). In both lineages, *M. stellatarum* was positively associated with the average style length and the average GS in the investigated populations, whereas *Hadena*, which only occurs in populations of RUPI, showed the opposite trend. The positive selection imposed by *M. stellatarum* is consistent with the general correspondence between pollinator proboscis length and floral tube length in specialized pollination systems (Alexandersson & Johnson, 2002; Bloch & Erhardt, 2008; Soteras et al., 2020). Conversely, *Hadena* moths, which use *Dianthus* flowers for oviposition (Prieto‐Benitez et al., 2017) lead to reduced floral structures in our study system. This finding aligns with previous studies documenting that flowers with longer floral tubes are more attractive for oviposition of pre‐dispersal seed predators (e.g., Brothers & Atwell, 2014; Cariveau et al., 2004). Therefore, producing smaller flowers may represent an advantage in populations experiencing high levels of predation by *Hadena*. This hypothesis aligns with our estimates of reproductive success at the individual level which confirmed that *Hadena* exerts strong negative selection on style length within the RUPI lineage, counteracting pollinator‐mediated selection and contributing to the observed phenotypic divergence between the two lineages. Importantly, this pattern is consistent with the among‐population trends observed in our study, where populations experiencing stronger *Hadena* pressure tend to show shorter styles and smaller GS (Figure 3), indicating that the direction of selection detected at the individual level aligns with the phenotypic and genomic divergence observed among populations. Also, these contrasting biotic pressures suggest that mutualistic and antagonistic interactions can jointly shape reproductive trait evolution and indirectly influence GS differentiation. In particular, in our study, these opposing biotic selective pressures appear to reinforce GS divergence between lineages (e.g., Cacho et al., 2021; Moreira et al., 2024). Indeed, the correspondence between the direction of selection and the existing GS differences between lineages suggest that divergent ecological selection may have amplified initial GS differences, potentially linked to genomic stress and transposable element activation commonly observed in populations colonizing volcanic islands (e.g., Craddock, 2016; Lanciano & Mirouze, 2018).

Overall, our study demonstrates that GS variation within *D. rupicola* reflects the combined effects of edaphic adaptation and divergent selection imposed by mutualistic and antagonistic biotic interactions. These results underscore that, in our study system, GS is not merely a neutral genomic attribute, but a trait indirectly shaped by ecological selection through its association with target traits. These results contribute to bridging the gap between micro‐ and macroevolutionary perspectives, suggesting that the ecological processes driving population divergence may also promote GS differentiation between closely related taxa. Also, our findings highlight the potential for ecological selection to drive genome evolution at early stages of divergence, linking microevolutionary ecological processes with long‐term diversification.

## AUTHOR CONTRIBUTIONS

Lucrezia Laccetti and Giovanni Scopece conceived the study. Lucrezia Laccetti and Antonia Cristaudo contributed to seed collection and floral data collection in natural populations and in the common garden. Emilio Petrone‐Mendoza led the collection of leaf functional traits data. Donata Cafasso and Fabio Pinheiro designed and performed genome size data collection. Lucrezia Laccetti conducted formal statistical analyses. All authors contributed to the interpretation of the results. Giovanni Scopece acquired funds to support the study. Lucrezia Laccetti and Giovanni Scopece wrote the first version of the manuscript. All authors commented on the original version and approved the final version of the manuscript.

## CONFLICT OF INTEREST STATEMENT

The authors declare no conflicts of interest.

## Supporting information


Appendix S1:

## Data Availability

Data (Laccetti, 2026) are available in Figshare at https://doi.org/10.6084/m9.figshare.30814790.v1.
