# Supplementary material for: Linking genome size variation to phenotypic selection on target traits
Source: Ecology. 2026 Jun 16;107(6):e70442. doi: 10.1002/ecy.70442 (PMC13270781; doi:10.1002/ecy.70442)
Supplement: Supplementary file 1 — Appendix S1: [file ECY-107-e70442-s001.pdf]

Appendix S1

Linking genome size variation to phenotypic selection on target traits

Lucrezia Laccetti, Emilio Petrone-Mendoza, Donata Cafasso, Antonia Cristaudo, Fabio Pinheiro, Giovanni Scopece

Ecology

Supplementary Tables

Table S1. Geographic locations of the 11 populations of *Dianthus rupicola* sampled in this study.

Population	Code	Lineage	Latitude	Longitude
**Lipari**	LIP	AEOL	38.4877	14.9313
**Milazzo**	MIL	AEOL	38.2697	15.2279
**Palinuro**	PAL	AEOL	40.0313	15.3089
**Salina**	SAL	AEOL	38.5508	14.8677
**Stromboli**	STR	AEOL	38.8092	15.2318
**Castelmola**	CAS	RUPI	37.8597	15.2777
**Cava Ispica**	CIS	RUPI	36.8378	14.9109
**Maratea**	MAR	RUPI	39.9923	15.7021
**Scilla**	SCI	RUPI	38.2508	15.7093
**Scopello**	SCO	RUPI	38.0827	12.8974
**Torre delle Mandre**	TMA	RUPI	38.0262	13.5988

Table S2. List of climatic variables extracted from the 11 populations of *Dianthus rupicola* investigated in the study.

Code	Variable description
**BIO1**	Mean Annual Temperature (MAT)
**BIO10**	Mean Temperature of Warmest Quarter
**BIO12**	Annual Precipitation (AP)
**BIO16**	Precipitation of Wettest Quarter (PWQ)
**BIO17**	Precipitation of Driest Quarter (PDQ)
**ELEV**	Elevation

Table S3. Outputs of generalized linear mixed models (GLMMs) testing phenotypic differences between two lineages of *Dianthus rupicola.* Significant associations are highlighted in bold.

**Floral trait**	χ^2	**df**	***P***
Number of flowers per inflorescence	**93.497**	**1**	**<0.001*****
Style length (cm)	**98.263**	**1**	**<0.001*****
Calyx length (cm)	**130.74**	**1**	**<0.001*****
Petal length (cm)	**11.307**	**1**	**<0.001*****
Petal width (cm)	3.217	1	0.073
Corolla diameter (cm)	**10.934**	**1**	**<0.001*****
Leaf mass per area (LMA; g/m^2)	0.285	1	0.594
Leaf area (cm^2)	**8.182**	**1**	**0.004****
Epidermal cell area (cm^2)	1.149	1	0.284
Stomata area (cm^2)	0.021	1	0.886

Table S4. Outputs of the Spearman Rank correlation analyses (*ρ)* between genome size and phenotypic traits in two lineages of *Dianthus rupicola* (AEOL and RUPI). Significant correlations are highlighted in bold.

**Phenotypic trait**	**Lineage**	***ρ***	**t (df)**	***P***
Number of flowers per inflorescence	AEOL	0.307	1.71 (28)	0.100
Number of flowers per inflorescence	RUPI	0.190	1.13 (34)	0.267
Style length (cm)	AEOL	**0.708**	**5.31 (28)**	**<0.001*****
Style length (cm)	RUPI	**0.578**	**4.11 (34)**	**<0.001*****
Calyx length (cm)	AEOL	0.232	1.26 (28)	0.218
Calyx length (cm)	RUPI	0.304	1.86 (34)	0.071
Petal length (cm)	AEOL	-0.201	-1.09 (28)	0.285
Petal length (cm)	RUPI	0.281	1.71 (34)	0.096
Petal width (cm)	AEOL	0.024	0.13 (28)	0.897
Petal width (cm)	RUPI	-0.236	-1.42 (34)	0.165
Corolla diameter (cm)	AEOL	-0.201	-1.09 (28)	0.285
Corolla diameter (cm)	RUPI	0.131	1.18 (34)	0.445
Leaf mass per area (LMA; g/m^2)	AEOL	-0.263	-0.98 (13)	0.343
Leaf mass per area (LMA; g/m^2)	RUPI	-0.292	-1.18 (15)	0.256
Leaf area (cm^2)	AEOL	-0.222	-0.82 (13)	0.427
Leaf area (cm^2)	RUPI	-0.104	-0.40 (15)	0.692
Epidermal cell area (cm^2)	AEOL	**0.589**	**2.53 (12)**	**0.026***
Epidermal cell area (cm^2)	RUPI	**0.805**	**4.90 (13)**	**<0.001*****
Stomata area (cm^2)	AEOL	**0.567**	**2.48 (13)**	**0.027***
Stomata area (cm^2)	RUPI	**0.551**	**2.56 (15)**	**0.022***

Table S5. Mean percentage of predation by *Hadena* species recorded in 11 populations of *Dianthus rupicola* across three consecutive flowering seasons.

Population	Lineage	*Hadena* predation (%)		
		**2020**	**2021**	**2022**
LIP	AEOL	0.00	0.00	0.00
MIL	AEOL	0.00	0.00	0.00
PAL	AEOL	0.00	0.00	0.00
SAL	AEOL	0.00	0.00	0.00
STR	AEOL	0.00	0.00	0.00
CAS	RUPI	25.45	35.19	28.21
CIS	RUPI	0.00	0.00	0.00
MAR	RUPI	0.00	0.00	0.00
SCI	RUPI	21.00	28.90	20.24
SCO	RUPI	5.07	8.86	14.04
TMA	RUPI	NA	NA	23.53

Table S6. Outputs of generalized linear mixed models (GLMMs) testing the association between biotic and abiotic factors, and phenotypic traits in two lineages of *Dianthus rupicola* (AEOL and RUPI). Significant associations are highlighted in bold.

Phenotypic trait	Predictor	Lineage	χ^2	df	*P*
Epidermal cell area (cm^2)	PC1	AEOL	0.947	1	0.331
Epidermal cell area (cm^2)		RUPI	0.138	1	0.710
Stomata area (cm^2)	PC1	AEOL	2.222	1	0.155
Stomata area (cm^2)		RUPI	0.044	1	0.834
Epidermal cell area (cm^2)	PC2	AEOL	0.676	1	0.411
Epidermal cell area (cm^2)		RUPI	2.075	1	0.150
Stomata area (cm^2)	PC2	AEOL	0.013	1	0.907
Stomata area (cm^2)		RUPI	1.306	1	0.253
Epidermal cell area (cm^2)	PC3	AEOL	0.162	1	0.688
Epidermal cell area (cm^2)		RUPI	**11.110**	**1**	**<0.001*** **
Stomata area (cm^2)	PC3	AEOL	**188.50**	**1**	**<0.001*** **
Stomata area (cm^2)		RUPI	**25.692**	**1**	**<0.001*** **
Style length (cm)	*Macroglossum stellatarum*	AEOL	**11.180**	**1**	**<0.001*** **
Style length (cm)		RUPI	**7.381**	**1**	**0.007** **
Style length (cm)	*Hadena* spp.	AEOL	NA	NA	NA
Style length (cm)		RUPI	**34.186**	**1**	**<0.001*** **

Table S7. Structural equation models assessment using bootstrap procedure with 1,000 iterations for the two lineages of *Dianthus rupicola* (RUPI and AEOL).

		**Standard bootstrap results**			**Percentile bootstrap quantiles**		**Result**	**R^2**
**Lineage**	**Effect**	$\hat{\beta}$	**Standard Error**	***P***	**5%**	**95%**		
RUPI	Style length → Seed predation (+)	0.586	0.066	<0.001	0.458	0.715	Supported	0.34
	Seed predation → Seed set (-)	-0.311	0.079	<0.001	-0.465	-0.157	Supported	0.26
	Style length → Seed set (-)	-0.260	0.086	0.003	-0.429	-0.091	Supported	
AEOL	Style length → Seed set (+)	0.497	0.096	<0.001	0.309	0.686	Supported	0.25

Supplementary Figures

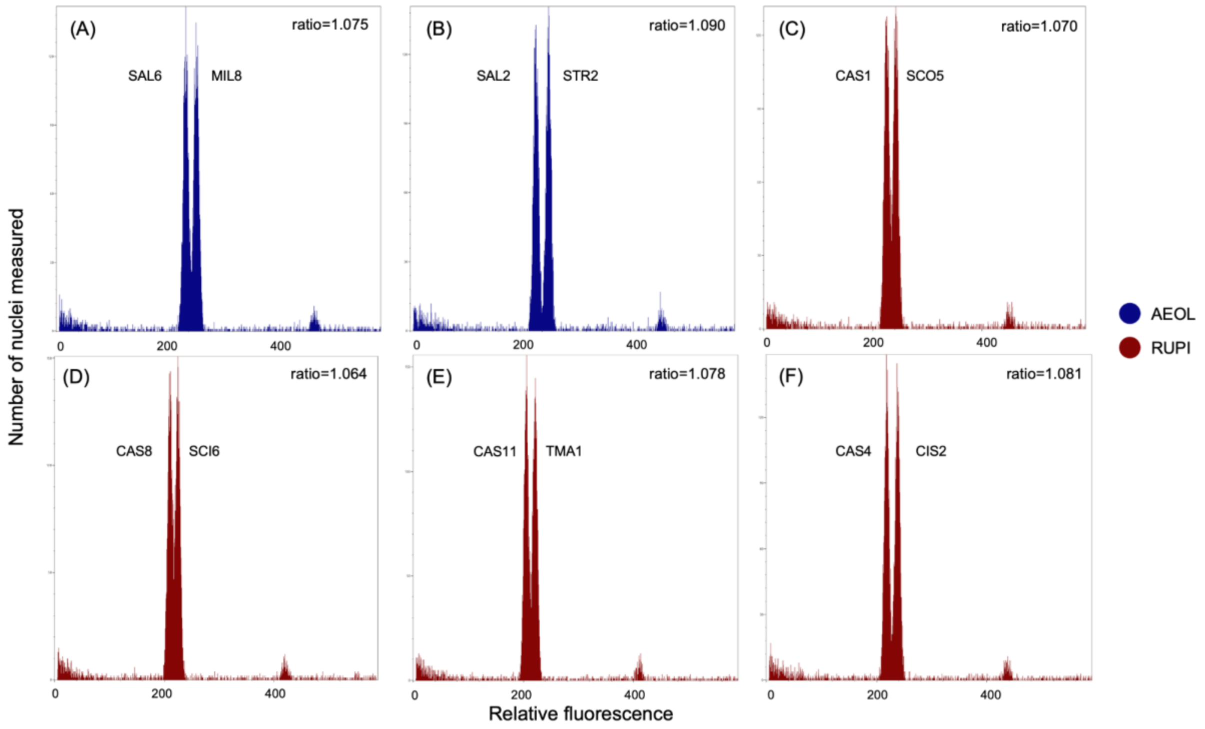

Figure S1. Differences in genome size in *Dianthus rupicola* individuals from the same lineage, as evidenced by double-peaks or bimodal peaks in flow cytometry histograms. Results were obtained by simultaneous measurements of two contrasting samples (A: SAL vs MIL; B: SAL vs STR; C: CAS vs SCO; D: CAS vs SCI; E: CAS vs TMA; F: CAS vs CIS). Population codes are listed in Table S1. Blue denotes the AEOL lineage, red denotes the RUPI lineage.

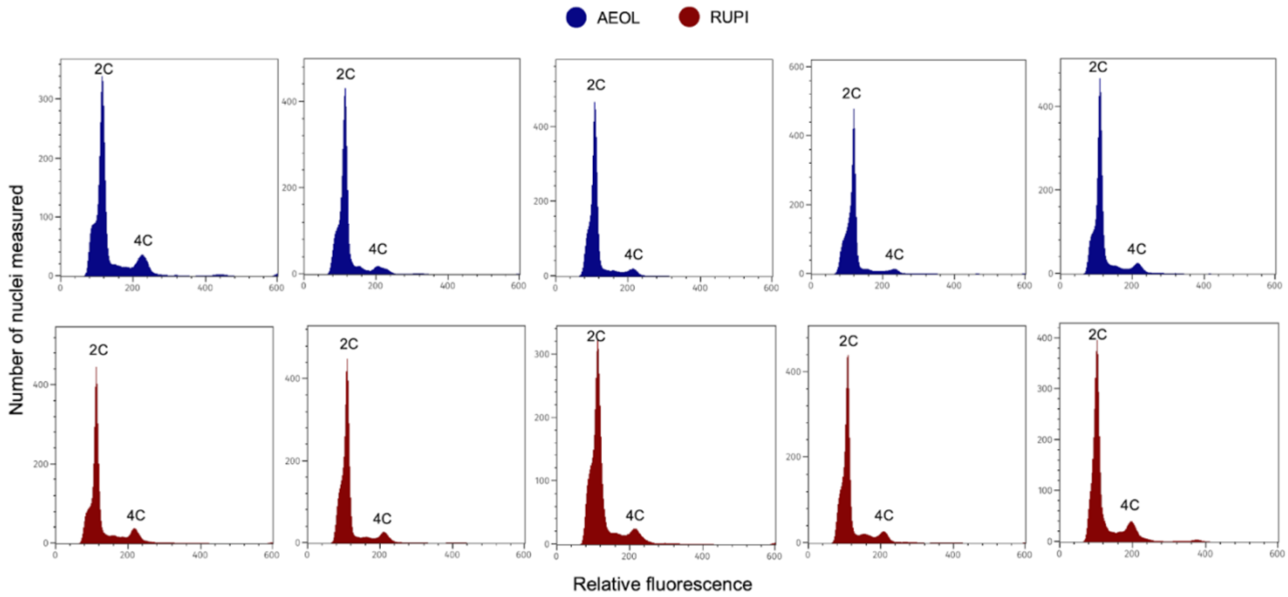

Figure S2. Tests of potential endopolyploidy in leaf tissues of the two lineages of *Dianthus rupicola* (AEOL and RUPI). Blue denotes the AEOL lineage, red denotes the RUPI lineage.

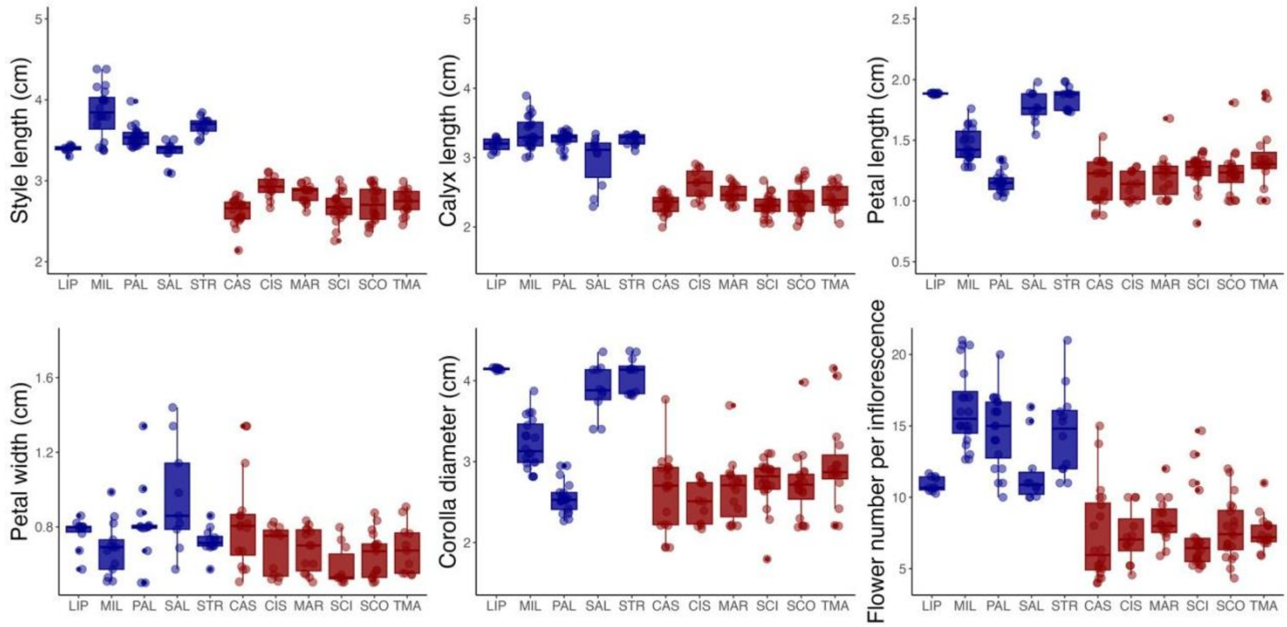

Figure S3. Variation in floral traits among *Dianthus rupicola* populations of two different lineages (AEOL and RUPI). Blue denotes the AEOL lineage, red denotes the RUPI lineage.

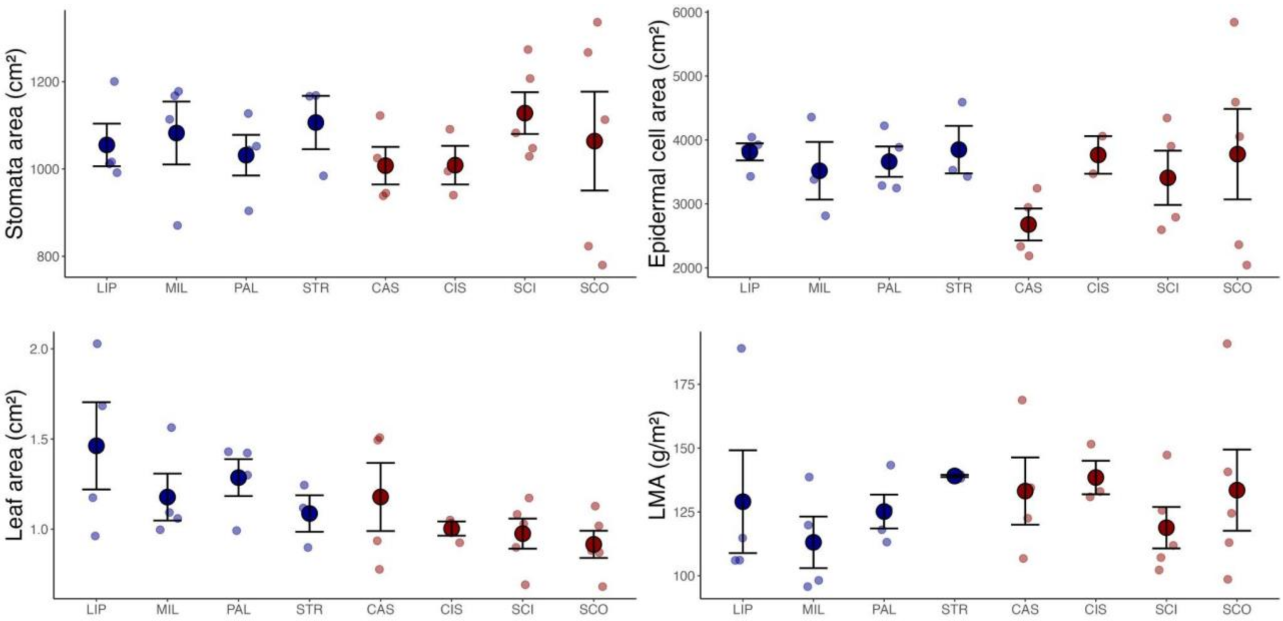

Figure S4. Variation in leaf functional traits among *Dianthus rupicola* populations of two different lineages (AEOL and RUPI). Blue denotes the AEOL lineage, red denotes the RUPI lineage.

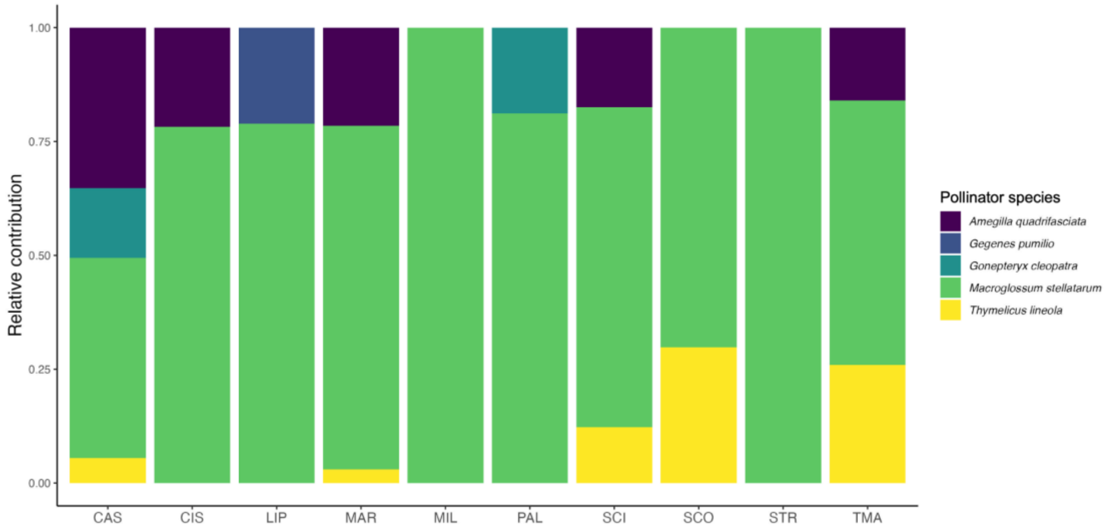

Figure S5. Pollinator assemblage of 10 populations of *Dianthus rupicola.*

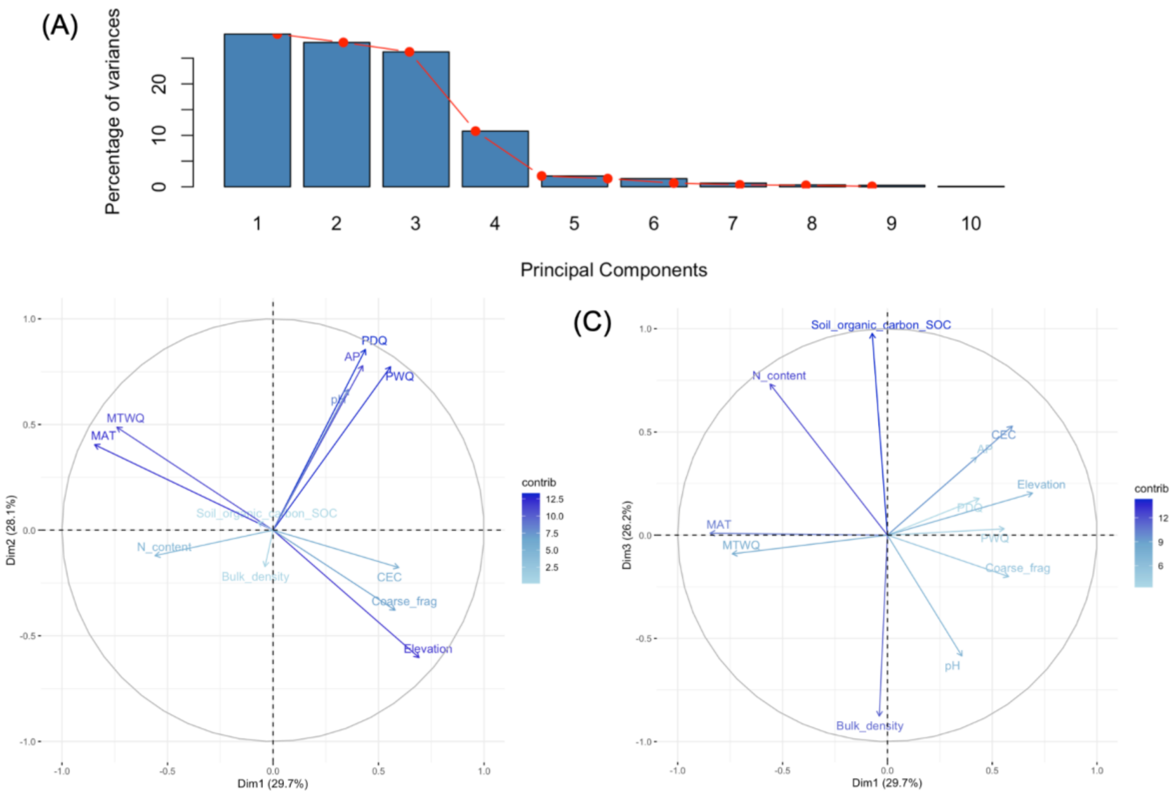

Figure S6. Principal component analysis (PCA) on abiotic factors experienced by *Dianthus rupicola* wild populations.

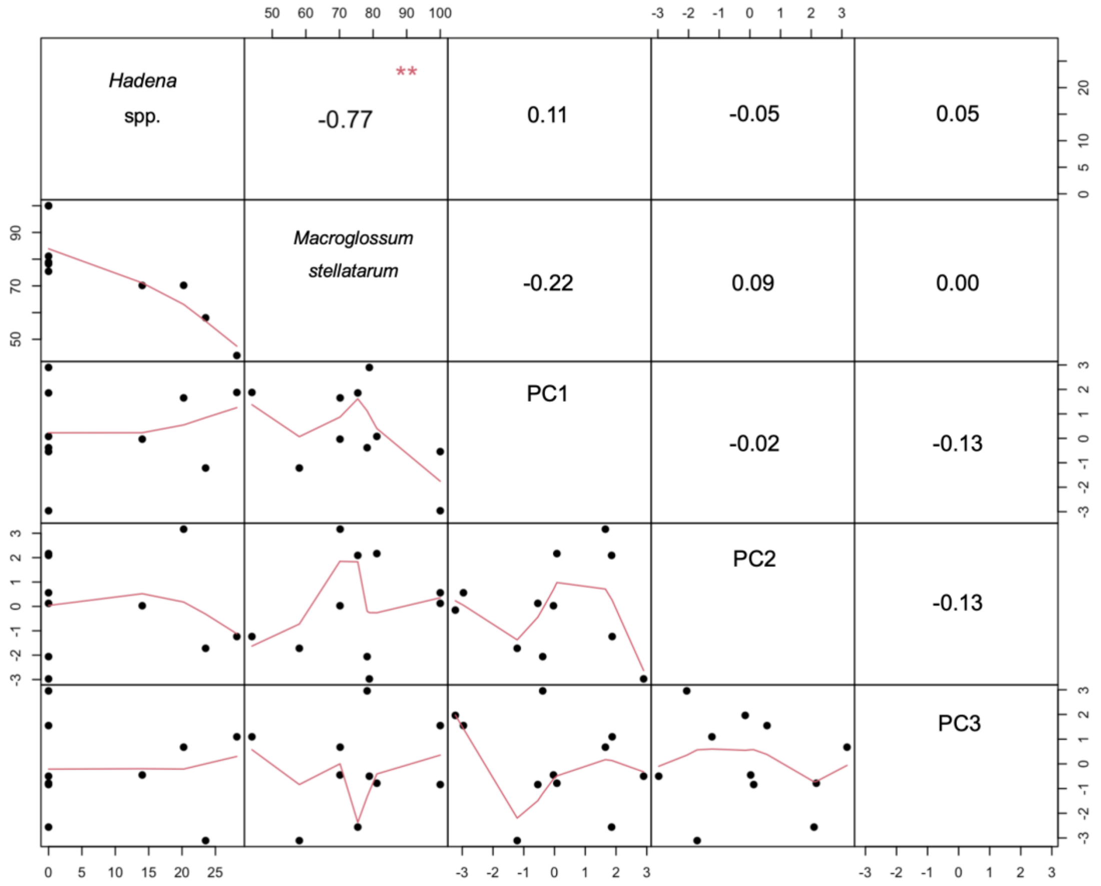

Figure S7. Kendall correlation between biotic and abiotic factors experienced by *Dianthus rupicola* wild populations. '**' $0.001 < P \leq 0.01$.
